# Bioavailability and -accessibility of subsoil allocated ^33^P-labelled hydroxyapatite to wheat under different moisture supply

**DOI:** 10.1038/s41598-020-74225-3

**Published:** 2020-10-13

**Authors:** Jan Wolff, Diana Hofmann, Maximilian Koch, Roland Bol, Andrea Schnepf, Wulf Amelung

**Affiliations:** 1 Institute for Crop Science and Resource Conservation (INRES)-Soil Science and Soil Ecology, University of Bonn, Nussallee 13, 53115 Bonn, Germany; 2 Institute for Bio- and Geosciences-IBG-3: Agrosphere, Forschungszentrum Jülich GmbH, 52425 Jülich, Germany

**Keywords:** Element cycles, Element cycles, Element cycles, Agroecology, Agroecology

## Abstract

Information on the bioavailability and -accessibility of subsoil phosphorus (P) and how soil moisture affects its utilization by plants is scarce. The current study examined whether and to which degree wheat acquires P from subsoil allocated hydroxyapatite and how this could be affected by soil moisture. We investigated the ^33^P uptake by growing wheat in two rhizotron trials (soil and sand) with integrated ^33^P-labelled hydroxyapatite hotspots over a period of 44 days using digital autoradiography imaging and liquid scintillation counting. We applied two irrigation scenarios, mimicking either rainfall via topsoil watering or subsoil water storage. The plants showed similar biomass development when grown in soil, but a reduced growth in sand rhizotrons. Total plant P_(tot)_ stocks were significantly larger in plants grown under improved subsoil moisture supply, further evidenced by enhanced P stocks in the ears of wheat in the sand treatment due to an earlier grain filling. This P uptake is accompanied by larger ^33^P signals, indicating that the plants accessed the hydroxyapatite because subsoil irrigation also promoted root proliferation within and around the hotspots. We conclude that even within a single season plants access subsoil mineral P sources, and this process is influenced by water management.

## Introduction

Phosphorus (P) is an essential though scarcely bioavailable macronutrient for plant growth^1,2^. As pointed out by Cakmak^3^, about 67% of world’s agricultural soils are even affected by P-deficiency. In soils, P originates from the weathering of rock material that becomes scarcer as pedogenesis progresses^4^. One major P containing mineral is apatite^5^, which may still represent a major primary P source in subsoils. Plants may acquire this P source when added to surfaces soils^6^, however, little is known on how crops access this mineral P form from the subsoil.

Phosphorous may be heterogeneously distributed in soils, with declining concentrations from the surface or near-surface horizons to the subsoil^7–10^. In the subsoil, the weathering rates of apatite and other P containing minerals is assumed to be low and hardly exceeding 5 kg ha^−1^ year^−1^, thus conflicting the general idea of subsoils becoming a main source of plant P nutrition^11,12^. Indeed, even if a small proportion of the mineral-bound total subsoil P is released as plant-available inorganic orthophosphate (P_i_)^11,12^, these phosphate ions may re-bind rapidly to other reactive soil constituents such as Fe- and Al-oxides as well as excessive Ca^2+^ ions^7,13^. These reactions immobilize the otherwise available orthophosphate P^7,9,12^, i.e., plants must develop specific P acquisition strategies to access subsoil P sources to overcome its limited bioavailability^13,14^.

A main parameter that triggers root growth and thus related uptake of (sub)soil nutrients is soil moisture^15–17^. In general, the diffusivity of P ions increases with increasing soil moisture supply^18^. A study by Clarke et al.^19^ demonstrated that the total P uptake of wheat was proportional to the contents of available water under field conditions. He et al.^20^ demonstrated in pot experiments that improved water availability also improved P uptake, while, e.g., Gutiérrez-Boem and Thomas^21^ only found a dependency of P uptake to the amount of P added. Finally, Wang et al.^22^ stated that with increasing dryness in the topsoil, crops become increasingly dependent on water from deeper soil horizons and, thus, may additionally increase the importance of the subsoil P for their supply. Apparently, managing soil moisture can be a key driver for improved P uptake from the subsoil^23^. As soil moisture may vary with soil depth, we placed hydroxyapatite, i.e., a mineral in which P is not directly bioavailable, in different soil depths, assuming that plants can adjust their root activity along patches with higher nutrient contents, eventually even showing tendencies of root proliferation in response to such localized nutrient reservoirs^24,25^.

To quantitatively track or even image the uptake of P into plants, experiments using radioactive isotopes (^32^P/^33^P) have been carried out with increasing frequency in the recent past^16,26^. These methods have the advantage that P can be reliably traced from specifically labelled P pools, since plants do not discriminate between the different P isotopes^27^. Therefore, the use of ^33^P labeled hydroxyapatite in rhizotron experiments may provide insights into the uptake of P from subsoil apatite minerals and the influence of soil moisture on this complex process. The aims of this study were (1) to analyze whether and to which degree an annual crop such as wheat is able to acquire P from hydroxyapatite located in subsoil, and (2) to determine the influence of different soil moisture contents on the uptake of P from this mineral source. To test these questions, rhizotron experiments with subsoil of either unfertilized arable soil or quartz sand as a P-free variant, were performed. The rhizotrons each contained two packages of ^33^P-labeled hydroxyapatite^28^, and the experiments were run under two irrigation scenarios. We then monitored the uptake of apatite-derived ^33^P distributed into the soil as well as its translocation within wheat plants using digital autoradiography imaging as well as liquid scintillation counting (LSC) and inductively coupled plasma–mass spectrometry (ICP–MS).

## Materials and methods

### Soil characteristics and treatment

As topsoil, the top 5 cm of a P-depleted Cambisol of the experimental research station of the University of Bonn at the Dikopshof (50° 48″ N, 6° 57″ E) was used. The soil was collected from a control plot that received no P-fertilization since 1931. The soil is completely decalcified with a silty loamy texture. For the subsoil, we used a composite sample from 45 to 75 cm soil depth collected at the experimental research station of the University of Bonn at Campus Klein-Altendorf. The subsoil was a decalcified Luvisol with a silty loamy texture and previously was shown to participate in the P nutrition of crops^29^. Both top- and subsoil were dried at 40 °C and then sieved to < 2 mm before usage. For the rhizotrons filled with sand, quartz sand (RBS, Inden, Germany) was used, which did not contain any plant available N, P, K and C^30,31^.

### Preparation of ^33^P-labeled hydroxyapatite hotspots

^33^P-labeled hydroxyapatite powder was prepared according to Wolff et al.^28^. The procedure takes about 30 h preparation time. Due to the restricted half-life of ^33^P (T_1/2_ = 25.35 d), the synthesis was started one day prior to the setup of the rhizotrons. As reaction conditions, 25 °C reaction and 100 °C calcination temperatures were used. For the synthesis of the hydroxyapatite a total ^33^P activity of 295 MBq was used which is equivalent to 26.4 MBq/g. It was filled into 7.5 × 5 cm bags, made of synthetic coarse-meshed fabric (0.4 × 0.5 mm mesh size), ensuring unrestricted root accessibility and proliferation during the experiment. For each bag, 70 g of subsoil or sand, remoistened to the water holding capacity (WHC) according to the surrounding soil with deionized water (see description below), was homogenized with 350 mg ^33^P labeled hydroxyapatite for 3 h using an overhead shaker. This amount of hydroxyapatite was chosen to represent a distinct nutrient hotspot with two times the amount of P in comparison to the surrounding soil as well as being sufficient for the detection of radioactive ^33^P after the full duration of the experiment.

### Preparation of the rhizotrons

**Figure 1:**
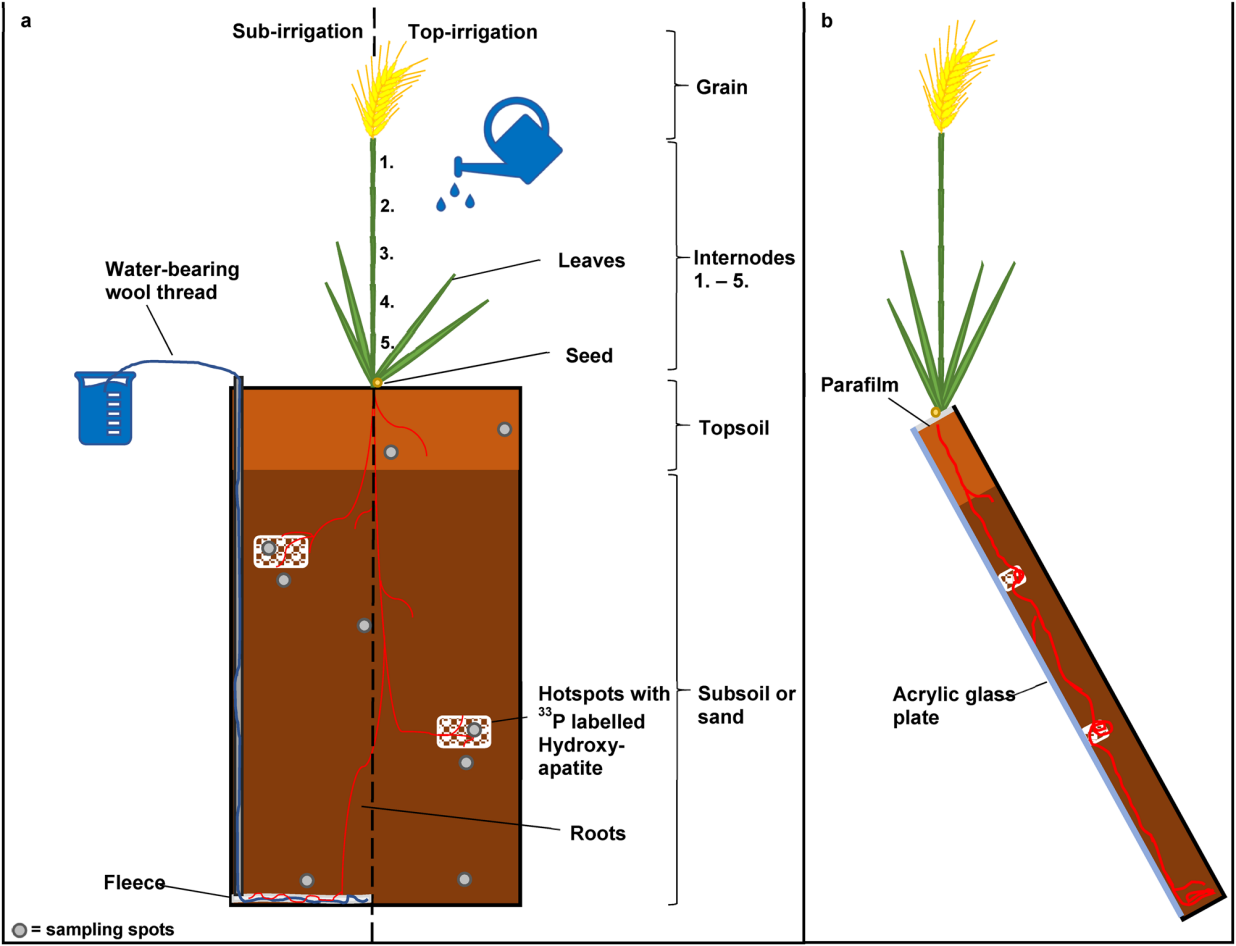
Setup of the rhizotrons displayed with the two irrigation systems used: (**a**) left = sub-irrigation, right = top-irrigation. Depicted are the different plant parts of the wheat and the sampling spots within the topsoil, subsoil and sand which were used for further analysis; (**b**) rhizotrons arranged at an angle of 45° showing the hydroxyapatite hotspot placement near the acrylic glass plate.

The rhizotrons used in these experiments were 16 box-like black polyvinyl chloride (PVC) corpuses (50 × 30 × 3.5 cm, inner diameter each), which were closed with transparent acrylic glass plates. A total of four different rhizotron scenarios were tested in quadruplicates each (n = 4): P poor subsoil with (1) top-irrigation vs. (2) sub-irrigation; quartz sand with (3) top-irrigation vs. (4) sub-irrigation (Fig. 1a). For the experimental approaches, in which sub-irrigation was intended, a cotton fleece was installed on the rhizotron floor, into which a wool thread was embedded. Plastic tubes were attached to the inner side walls of the rhizotrons, through which the wool threads (1 m on each side) were led from the fleece to the upper edges of the rhizotrons. These woolen threads were connected to water-filled glass bottles on both sides of the rhizotron, creating a water-bearing link to the fleece, and thus to the subsoil (Fig. 1a, left side). Top-irrigation was performed with tap water using a garden sprayer.

Rhizotrons with top-irrigation were initially set to 75% of the maximum WHC in the top- and subsoil. Rhizotrons with sub-irrigation via the fleece system were set to 50% WHC in the topsoil and 75% WHC in the subsoil in order to create an increasing moisture gradient from the top- to the subsoil. Subsequently, rhizotrons with subsoil will be described as “soil rhizotrons”, those with quartz sand as subsoil equivalent will be describes as “sand rhizotrons”. The total amount of tap water used for irrigation was similar for both top- and sub-irrigation trials.

The remoistened subsoil or sand was filled layer by layer up to 40 cm into the rhizotrons with a self-made pestle to obtain a bulk density of 1.4 g cm^−3^. In 20 cm and 30 cm total depth of the rhizotron, one bag pre-filled with ^33^P-labelled hydroxyapatite was implemented as P hotspot near the acrylic glass plate. The upper 10 cm of the rhizotrons were filled up with remoistened topsoil at a bulk density of 1.1 g cm^−3^. Soil physical parameters were monitored throughout the experiment using moisture sensors and dielectric porous matric potential sensors (MPS2; Meter Environment, München, Germany). One sensor of each type was placed in the top- and subsoil of each soil treatment.

### Plant cultivation and climate chamber settings

Summer wheat seeds (*Triticum aestivum* L. cv. Cornetto, KWS Saat SE, Einbeck, Germany) were germinated on moistened filter paper in a petri dish. At the beginning of the experiment, the seedlings were selected to ensure a uniform stage of development, with one seedling per rhizotron being carefully planted in the center of the topsoil. The upper opening of the rhizotrons were then covered with parafilm, except an opening for plant growth, to minimize transpiration during the experiment. The prepared rhizotrons were lined up at a 45° angle in a climate chamber with the transparent plate facing downwards, so that root growth could be visually observed via the acrylic glass plates (Fig. 1b). The experimental duration was 44 days within the climate chamber, with a 16/8 h change of day/night. The average daytime temperature was 20 ± 5 °C supported by a UV lamp with an intensity of 320 µmol m^−2^ s^−1^, while the temperature dropped to 15 ± 3 °C during nighttime. No control of air humidity was set. The wheat plants of all rhizotrons were irrigated via the topsoil until the roots were about to reach the subsoil or sand in order to prevent an early drying of the plants.

### ^33^P imaging via digital autoradiography

After the 44-day experimental period, all rhizotrons were opened and the distribution of the ^33^P tracer within the plants and substrates of each treatment was investigated by digital autoradiography prior to plant removal, though still lacking quantitation subroutines as simultaneously developed by Koch et al.^26^. After the end of the experiment, the rhizotrons were placed on a table and the acrylic glass plates were carefully removed to prevent any mechanical impact on the soil or roots. Afterwards, phosphor imaging plates (Resolution image plate, DÜRR NDT GmbH, Bietigheim-Bissingen, Germany), which were wrapped in thin plastic foils to prevent damage or contamination, were carefully placed on the plants or on the soil or sand, therewith covering the whole rhizotron. During an exposure time of 18 h in complete darkness, the use of broad, heavier plates placed onto the imager plates ensured their full contact with the plants and rhizotrons. Due to their light sensitivity, the imager plates were scanned in the dark using a plate scanner (Bioimager CR35 Bio, Raytest, Straubenhardt, Germany) in sensitivity mode at a resolution of 100 µm. The resulting digital images were further processed using the evaluation software AIDA (AIDA Image Analyzer 2D densitometry program, Raytest, Straubenhardt, Germany).

### Plant, soil, and statistical analyses

After finishing the digital autoradiography of all rhizotrons, the wheat plants, including their roots, were removed, divided into different segments (ears, internodes of the stems, leaves, seeds, roots) and dried at 40 °C for 24 h. Following this, dry weights were determined and plant parts were ground for further analysis. Total elemental concentration of phosphorus (P), calcium (Ca), magnesium (Mg) and potassium (K) of all plant parts, hotspots and, to cover the whole soil column, several spots of the topsoil and subsoil/sands were analyzed by inductively coupled plasma – mass spectrometry (7900 ICP-MS, Agilent Technologies International Japan Ltd., Tokyo, Japan) after acid digestion (65% HNO_3_ and 37% HCl). Soil pH was determined at a soil:solution ratio of 1:2.5 using 0.01 mol L^−1^ CaCl_2_. For radioactive P analyses, a subset of the dried plants, roots, hotspots and soils/sands of each treatment were also pressure digested as described in Bauke et al.^16^ by 6 h heating at 180 °C (0.5 g of each sample, dissolved in 4 mL concentrated nitric acid). Subsequently, an aliquot of the resulting solution was then mixed with 10 mL Ultima Gold XR scintillation cocktail (PerkinElmer Inc., USA) to improve the counting efficiency. The ^33^P activity was analyzed in triplicate using liquid scintillation counting (LSC). The ^33^P data were calculated back to the beginning of the experiment through consideration of radioactive decay following Eq. (1) with λ representing the decay constant. This allowed a reconstruction of the total amount of ^33^P being taken up by the wheat plants at the end of the experiment.

1 $$T_{\frac{1}{2}} = \frac{ln2}{\lambda }$$

Graphs, curves and statistical analyses were performed using SigmaPlot (version 13.0, Systat Software Inc.). Normal distribution was tested using Shapiro–Wilk tests and homogeneity of variance of the data was tested using the Brown-Forsythe test. If a normal distribution of data was not given, a log transformation of the corresponding data was performed. Significant differences were tested using analysis of variance (ANOVA) combined with Holm-Sidak post hoc test for pairwise multiple comparison.

## Results and discussion

### Soil status, plant development and root architecture

**Figure 2:**
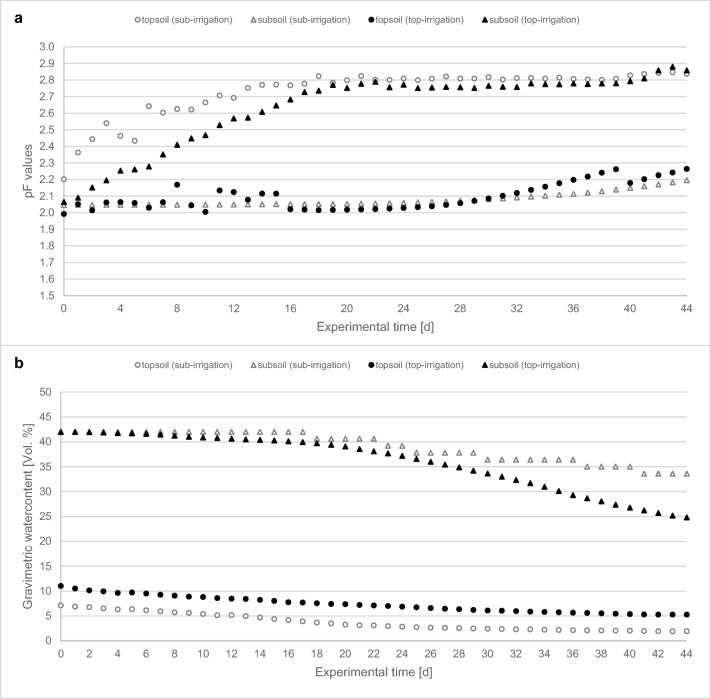
Changes of (**a**) pF Values and (**b**) gravimetric soil moisture contents in dependency of the specific bulk density (topsoil 1.1 g cm^3^; subsoil 1.4 g cm) plotted over time for the two soil rhizotron trials (grey = sub-irrigation; black = top-irrigation).

Measurements of the gravimetric soil water content of the subsoils in both variants of the soil rhizotrons showed clear differences depending on the irrigation scenario: For the variants with top-irrigation, the water availability in the topsoil corresponded to a pF value of 2.0 at the beginning of the experiment, while for the variants with sub-irrigation the pF value was 2.2 (Fig. 2).

The initial pF value of the subsoils was approximately 2.1 in both variants. Irrigation affected the time course of pF values: It remained within the range of the field capacity (pF 2.1–2.2 at day 44) in the irrigated top- and subsoils, respectively (Fig. 2a). The other, non-irrigated complementary soil layers dried out and the pF values increased to 2.8–2.9 from approximately day 20 onwards. Changes in gravimetric water content reflected these scenarios: gravimetric soil moisture remained at 5% in the irrigated topsoil but dropped to 2% (the matric potential declined by − 53 kPa) in the variants with subsoil irrigation. Also, the irrigation of the subsoil almost maintained a constant water content (the matric potential declined by − 5 kPa, only), while the subsoil dried out upon top-irrigation (the matric potential declined by − 61 kPa). Consequently, our setup allowed a comparison of plant growth and related P acquisition from soil with either sufficient water supply in top- or subsoil, respectively.

The 10 cm thick layer of topsoil, which was implemented in all rhizotron types, supported similar developments of wheat plants in all rhizotrons. Progressing plant developmental changes in both the aboveground plant parts and the root architecture were observed once the sand was accessed by roots: Since then, plant growth was significantly reduced in the sandy rhizotrons compared with those filled with soil as illustrated by the measured plant parameters after 44 days (Table 1, quantitatively evaluated only for the end of the experiment).

**Table 1:** Characteristics of plants, ^33^P uptake and water inputs due to the different forms of irrigation from different rhizotron trials (n = 3) after 44 days; different letters indicate significant differences among different rhizotron trials (p < 0.05). Displayed are mean values with standard deviations.

Rhizotron setup	Soil		Sand	
Irrigation	Subsoil	Topsoil	Subsoil	Topsoil
Av. wet weight (g)	9.0 ± 2.3 a	8.8 ± 1.5 a	1.7 ± 0.4 b	0.6 ± 0.2 b
Av. dry weight (g)	2.5 ± 0.2 a	2.0 ± 0.3 a	0.4 ± 0.1 b	0.2 ± 0.1 b
Av. Plant water content (%)	71.0 ± 6.5 a	77.2 ± 1.1 a	74.4 ± 7.7 a	60.3 ± 16.3 a
Av. plant height (cm)	58.8 ± 8.7 a	54.7 ± 4.4 a	39.3 ± 12.8 a	29.3 ± 7.6 b
Av. lengths of ears (cm)	7.85 ± 1.1 a	7.63 ± 1.1 a	4.83 ± 1.6 b	2.85 ± 0.5 c
Av. number of stems	3.5 ± 0.5 a	3 ± 1 ab	1 ± 0 b	1 ± 0 b
Av. amount of leaves	12.5 ± 1.3 a	12.25 ± 1.5 a	4.5 ± 0.6 b	4.75 ± 1.3 b
Av. number of internode	5 ± 0	5 ± 0	3 ± 0	3 ± 0
Av. number of ears	2 ± 0	2 ± 0	1 ± 0	1 ± 0
Irrigation quantity after 44 days (ml)	2526	2400	3242	2400

The root architecture within the sand rhizotrons was characterized by two thicker primary roots, which grew strictly towards the bottom of the rhizotrons without exploration of the remaining areas of the rhizotron (Fig. S1; Supplementary Information). The poor growth of the aboveground plant structures in combination with a pronounced root growth within both sand rhizotron types could be attributed to nutrient stress, as plant resources are allocated to root growth under nutrient stress and thus represent a significant metabolic cost factor^9,32^. As soon as the roots of the sub-irrigated sand rhizotrons reached the fleece, they started proliferating and rooting within it, thus increasing their water uptake as indicated by increased need for irrigation after 44 days (Table 1).

In all rhizotrons, roots reached the apatite hotspots. Nevertheless, only the roots of the rhizotron variants filled with soil as well as the sub-irrigated sand rhizotrons proliferated within the hydroxyapatite, thus prioritizing the deeper located hotspots (Fig. S2; Supplementary Information). A study of Lynch and Brown^9^ described this behavior as a reaction of the plant to P stress, which they try to overcome by excessive root proliferation within hotspots in the soil once they have encountered them. Noteworthy, this process was only pronounced upon sub-irrigation, with reduced water stress near the hydroxyapatite hotspots^8^. The water availability in the topsoil was of minor importance in this regard. In general, low availability of water can have a drastic effect on root physiology and, in combination with nutrient stress, it can exacerbate root costs^8^.

The root architecture of the soil rhizotrons showed a different pattern to those filled with sand: The entire soil column and the hotspots were interwoven with finer roots (Fig. S1, SI). These optical differences were confirmed by differences in the biomass development of the wheat plants. Many parameters, i.e., the weight (p = 0.006), total height (p < 0.001), length of the ears (p < 0.001), and number of stems, were significantly larger in the soil than in the sand rhizotrons (Table 1). In contrast, differences among the irrigation treatments were not apparent for the soil rhizotrons, whereas plant growth was additionally suppressed by top-irrigation of the sand rhizotrons (Table 1). With an average of 12.4 leaves per plant, the wheat from the soil rhizotrons, for instance, produced 63% more leaves than the wheat from the sand rhizotrons, also the weight to leaf ratios were 86% above those of the two sand rhizotrons variants. The results are consistent with previous studies showing that water stress may reduce leaf area while P deficiency may reduce the rate of leaf development, the number of simultaneous emerging leaves, and thus the final number of leaves^21,33,34^. In addition, the number and length of internodes also differed. While wheat plants from soil rhizotrons formed three longer internodes, wheat plants from sand rhizotrons formed five short internodes. This morphological characteristic in the sand rhizotrons is an additional indication of both nutrient and primary water stress, known to lead to a reduction in internodal elongation^35,36^.

### Nutrient content analyses

The total elemental concentrations of P, N, Ca, Mg and K within all plant parts and soils of the different rhizotron setups are shown in Table 2. In general, nutrient acquisition was most pronounced in the sub-irrigated soil rhizotrons, followed by the top-irrigated trials and the sub- and top-irrigated sand rhizotrons (Table 2).

**Table 2:** Mean values with standard deviations of total elemental concentrations of P, Ca, Mg and K in gram per kilogram of plants or soils from different rhizotron variants (n = 3 each). Different letters within the same row indicate significant differences among the trials (P < 0.05).

Treatments	Soil		Sand	
	Sub-irrigation (g kg^−1^)	Top-irrigation (g kg^−1^)	Sub-irrigation (g kg^−1^)	Top-irrigation (g kg^−1^)
**Elemental concentration**				
**P**				
Ears	2.91 ± 0.18 a	2.67 ± 0.20 a	6.96 ± 0.29 b	0.09 ± 0.05 c
Stems	1.83 ± 0.23 a	1.64 ± 0.34 a	0.77 ± 0.80 b	0.19 ± 0.23 c
Leaves	1.32 ± 0.18 a	1.29 ± 0.04 a	0.86 ± 0.07 b	0.50 ± 0.03 c
Seed	25.92 ± 4.40 a	6.99 ± 2.08 b	3.73 ± 1.18 c	0.42 ± 0.17 d
Roots	0.88 ± 0.64 a	1.20 ± 1.18 b	0.81 ± 0.21 a	0.08 ± 0.04 c
Topsoil	0.39 ± 0.02 a	0.35 ± 0.02 b	0.36 ± 0.01 ab	0.36 ± 0.02 ab
Apatite 20 cm	1.57 ± 0.13 a	2.26 ± 0.18 b	0.79 ± 0.07 c	0.82 ± 0.05 c
Apatite 30 cm	0.96 ± 0.04 a	0.75 ± 0.06 b	0.47 ± 0.09 c	0.65 ± 0.03 b
Subsoil	0.40 ± 0.01 a	0.39 ± 0.01 b	0.01 ± 0.00 c	0.01 ± 0.00 c
**Ca**				
Ears	0.86 ± 0.05 a	1.01 ± 0.12 a	7.68 ± 1.06 b	1.19 ± 0.68 a
Stems	0.89 ± 0.16 a	1.09 ± 0.26 a	7.17 ± 1.68 b	3.10 ± 1.47 c
Leaves	5.35 ± 0.45 a	5.00 ± 0.98 a	12.06 ± 3.90 b	11.55 ± 2.11 b
Seed	28.91 ± 14.03 ac	8.40 ± 3.02 bc	26.62 ± 5.54 ab	5.01 ± 0.84 c
Roots	7.16 ± 5.80 a	9.71 ± 7.32 a	2.77 ± 1.19 b	1.10 ± 0.86 b
Topsoil	3.17 ± 0.06 a	3.11 ± 0.16 a	3.18 ± 0.09 a	4.45 ± 0.33 b
Apatite 20 cm	5.25 ± 1.38 a	7.11 ± 1.69 a	248.66 ± 3.73 b	281.31 ± 5.01 c
Apatite 30 cm	4.49 ± 0.19 a	4.43 ± 0.29 a	242.41 ± 3.68 b	283.88 ± 1.94 c
Subsoil	4.00 ± 0.15 a	3.84 ± 0.16 a	254.46 ± 62.85 b	282.72 ± 6.42 c
**Mg**				
Ears	0.64 ± 0.04 a	0.68 ± 0.05 a	3.75 ± 0.50 b	0.70 ± 0.16 a
Stems	0.45 ± 0.06 a	0.57 ± 0.15 a	1.94 ± 0.34 b	0.90 ± 0.27 c
Leaves	1.08 ± 0.31 a	1.26 ± 0.28 a	4.05 ± 0.68 b	4.34 ± 1.65 b
Seed	13.88 ± 4.08 a	3.50 ± 2.17 b	17.71 ± 2.36 a	1.11 ± 0.15 b
Roots	5.23 ± 4.81 a	6.57 ± 6.67 a	5.14 ± 2.77 a	1.68 ± 1.67 a
Topsoil	0.39 ± 0.03 a	2.23 ± 0.06 b	2.21 ± 0.03 b	2.24 ± 0.03 b
Apatite 20 cm	4.18 ± 0.14 a	4.02 ± 0.09 a	3.74 ± 0.03 b	3.73 ± 0.02 b
Apatite 30 cm	4.17 ± 0.08 a	4.07 ± 0.12 a	3.71 ± 0.04 b	3.73 ± 0.09 b
Subsoil	4.22 ± 0.05 a	4.12 ± 0.10 a	3.78 ± 0.13 b	3.75 ± 0.20 b
**K**				
Ears	11.98 ± 0.88 a	12.48 ± 0.58 a	36.01 ± 6.87 b	3.94 ± 3.53 a
Stems	18.15 ± 3.42 ab	17.67 ± 4.06 ab	20.65 ± 12.56 a	7.37 ± 4.30 b
Leaves	14.53 ± 3.58 a	18.51 ± 3.77 a	9.46 ± 3.28 a	14.66 ± 5.19 a
Seed	252.45 ± 29.27 a	69.38 ± 18.42 b	36.48 ± 4.97 c	2.95 ± 0.45 c
Roots	12.21 ± 10.83 a	16.05 ± 18.23 b	0.82 ± 0.18 c	0.95 ± 0.24 c
Topsoil	4.51 ± 0.12 ab	4.56 ± 0.06 a	4.40 ± 0.13 ab	4.36 ± 0.01 b
Apatite 20 cm	7.67 ± 0.19 a	7.33 ± 0.15 b	0.37 ± 0.01 c	0.37 ± 0.01 c
Apatite 30 cm	7.69 ± 0.16 a	7.02 ± 0.30 b	0.39 ± 0.03 c	0.37 ± 0.01 c
Subsoil	7.69 ± 0.11 a	7.25 ± 0.24 b	0.39 ± 0.09 c	0.38 ± 0.01 c

The trend to improved development of the wheat plants in sub-irrigated treatments (Table 1) was thus frequently accompanied by elevated nutrient concentrations within the plants of the soil rhizotrons (Table 2), and thus by elevated nutrient stocks within the plant compartments (Table 3, see also discussion below). The data thus confirms that uptake of P strongly depends on soil moisture availability^20,37,38^. The acquired P was ultimately concentrated in the ears (Tables 2, 3), because stress induced by restricted nutrient and water availability leads to an increased shift of nutrients into the grains of ears^39^.

**Table 3:** P stocks of plant segments (n = 3) from different rhizotron trials. Different letters within one plant segment indicate significant differences among the setups (p < 0.05). Displayed are mean values with standard deviations.

Treatments	Soil		Sand	
	Sub-irrigation (mg)	Top-irrigation (mg)	Sub-irrigation (mg)	Top-irrigation (mg)
**P stocks**				
Ears	0.84 ± 0.15 a	0.75 ± 0.10 a	0.19 ± 0.01 b	0.01 ± 0.00 b
Stems	2.54 ± 0.44 a	1.75 ± 0.26 b	0.07 ± 0.03 c	0.03 ± 0.02 c
Leaves	1.08 ± 0.04 a	0.77 ± 0.14 a	0.04 ± 0.02 b	0.02 ± 0.00 b
Seed	0.35 ± 0.24 a	0.17 ± 0.06 a	0.11 ± 0.08 a	0.01 ± 0.00 a
Roots	0.49 ± 0.32 a	0.50 ± 0.05 a	0.09 ± 0.01 b	0.02 ± 0.02 c
Total	5.28 ± 0.46 a	3.95 ± 0.55 b	0.50 ± 0.14 c	0.12 ± 0.03 c

Noteworthy, the remaining P in the surface soil hardly contributed to increased uptake of nutrients from the sand rhizotrons. This indicates that the effort of finding nutrients and water in the subsoil was greater than the investment in a common strategy called “topsoil foraging”, which describes an increased root growth in the topsoil due to higher soil P accumulations^11,16,40^. We assume that the plants somehow recognized that the topsoil cannot provide sufficient plant-available P in the long-term, making an investment in greater depth more profitable.

The concentration patterns of Ca, Mg and K in the plants only partly resembled those of P, with Ca and K being partly enriched in some plant compartments of the sand rhizotrons relative to the soil rhizotrons (Table 2). These differences likely result from both different priorization by the plants and by different mobility of these nutrients within the plants^41,42^. The overall stock of these nutrients in the plants was lower in the sand rhizotrons, due to lower plant biomass development (Table S1, Supplementary Information). Differences in nutrient uptake were also reflected by remaining nutrient concentrations in the seeds. In the comparison of the two soil rhizotron variants, the concentrations of P, Ca, Mg and K in the seeds were about 71 to 75% larger in the sub-irrigated soil rhizotrons than in the top-irrigated ones; relative differences in the sand rhizotrons also being larger (Table 2), simply because utilization of nutrients from the seeds was less required at elevated plant growth conditions in the sub-irrigated trials.

Multiplying biomass weights with their P concentrations yielded the total P_(tot)_ contents per plant organ (Table 3). The data generally confirmed the information from the above-mentioned tables that P accrual was best in sub-irrigated soil rhizotrons, followed by the top-irrigated ones and the two sand rhizotron variants (Table 3). No significant difference could be determined between the sand rhizotron variants themselves. Leaves and stems acquired most P as intermediate storage organs. Our data is thus in line with observations of Wang et al.^22^ that higher root proliferation within the subsoil due to superior subsoil moisture conditions improves P uptake from the subsoil.

The P stocks in the ears contributed between 11 and 23% to the total shoot P, reflecting that the grain filling phase was not finished in the soil rhizotrons after 44 days, with grains becoming the major P-sink at maturity, containing up to 89% of the total shoot P^43^. For the sand rhizotrons, accumulation of P in the ears was more pronounced, reaching up to 63% of the total shoot P in the sub-irrigation trials and indicating a premature grain filling induced by stress. According to Raghothama^2^, this phenomenon can be related to P-starvation, which can significantly increase the uptake and translocation of P through the roots to the ears. Besides, this finding may support Gutiérrez-Boem and Thomas^21^, who hypothesized that P and water availability do not interact in regard to biomass production except when it comes to the allocation of nutrients within the plant.

### Distribution of ^33^P within plant and soil

**Figure 3:**
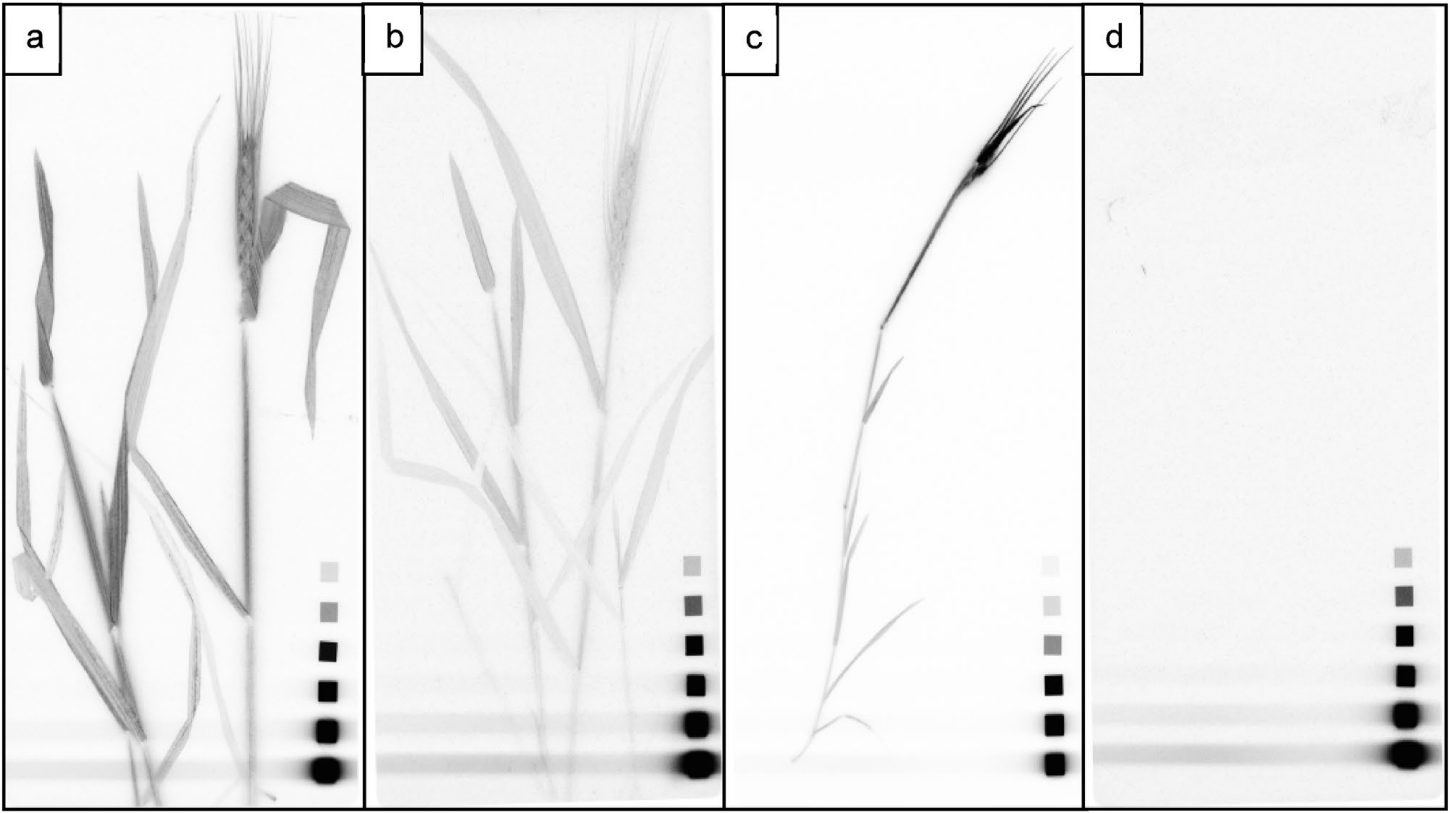
Digital autoradiography images from wheat plants of different rhizotron setups after 44 days; (**a**) (soil) and (**c**) (sand) rhizotrons with sub-irrigation; (**b**) (soil) and (**d**) (sand) rhizotrons with top-irrigation. The scales refer to ^14^C polymer references ranging from 66 to 18,450 Bq/cm^2^ (IPcal test source array; ELYSIA-Raytest, Straubenhardt, Germany), which allows conclusions to be drawn about qualitative differences of the ^33^P activities through the color gradations. The darker the lines, the larger the P uptake by the respective plant parts.

Digital autoradiography was used to analyze both the plants and the soil/sand. Due to the high ^33^P activity chosen at the beginning of the experiment, there was still a sufficient ^33^P activity to generate distinct images after 44 days (Fig. 3).

The images of the wheat plants supported our hypothesis that wheat plants were able to access and take up P from hydroxyapatite hotspots. Furthermore, they supported the conclusions of the different P uptake patterns drawn from the elemental analysis data. These visible differences in the P uptake patterns between the different rhizotron variants can be evaluated semi-quantitatively on the basis of the displayed intensities. The images of the plants from the two soil rhizotron variants indicated a fairly homogeneous distribution of the ^33^P in the plants, with higher activities in the plants grown with sub-irrigation (Fig. 3a,b). The respective plants from the sand rhizotrons confirmed the ^33^P accrual inside the ears, whereas in the sand rhizotrons with top-irrigation, little, if any, ^33^P was detected in the plants (Fig. 3c,d, respectively).

**Figure 4:**
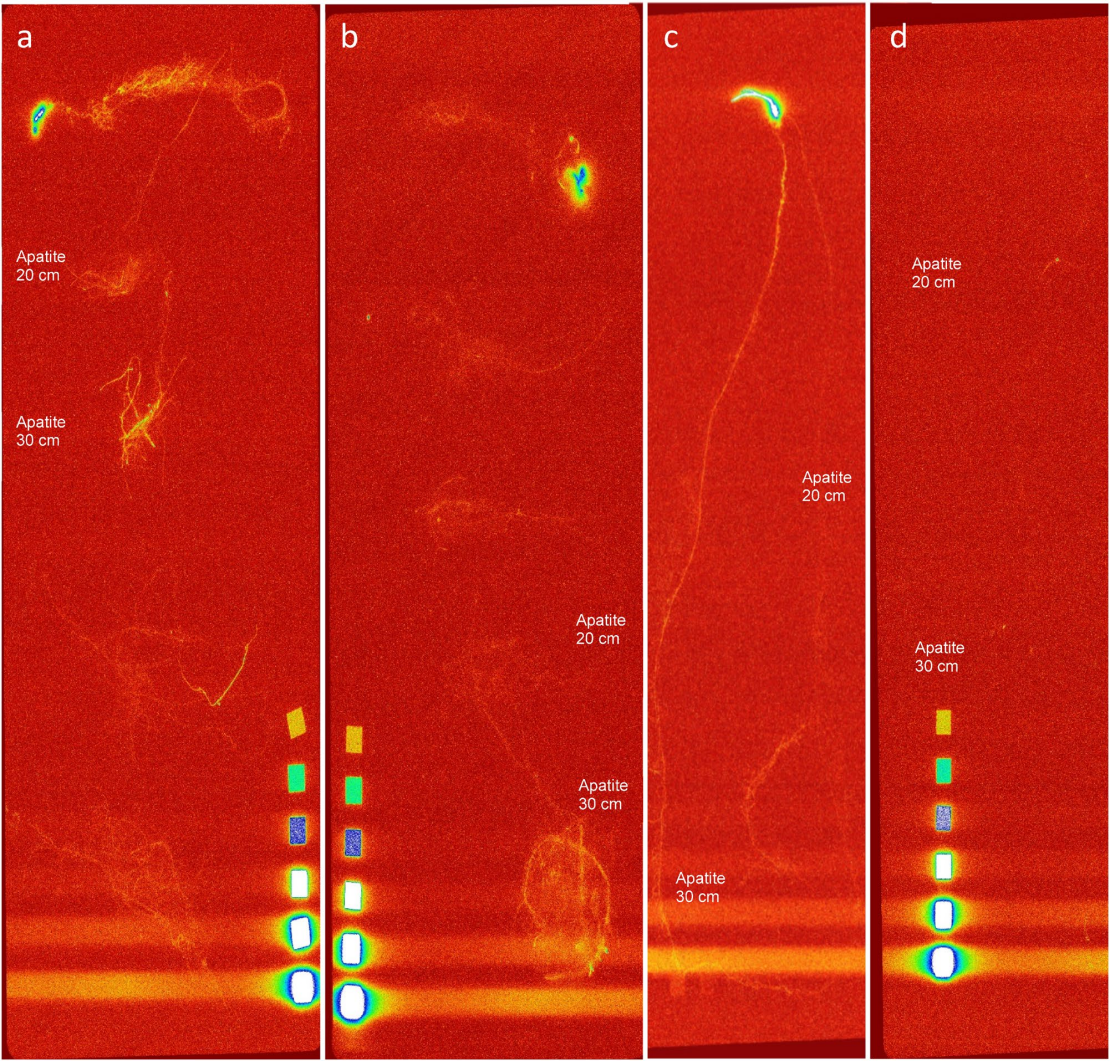
Digital autoradiography images of wheat roots with the seedling corn from different rhizotron setups after 44 days; (**a**) (soil) and (**c**) (sand) rhizotrons with sub-irrigation; (**b**) (soil) and (**d**) (sand) rhizotrons with top-irrigation. For image acquisition, the displayed roots were extracted from the rhizotrons before imaging for better visualization. Therefore the original ^33^P hydroxypatatite hotspots are not displayed in these images, but the places of these hotspots were marked according to the depth where the apatite was placed, i.e., at either 20 or 30 cm. The scales refer to ^14^C polymer references ranging from 66 to 18,450 Bq/cm^2^ (IPcal test source array; ELYSIA-Raytest, Straubenhardt, Germany), which allows conclusions to be drawn about qualitative differences of the ^33^P activities through the color gradations. The lighter the lines, the larger the P uptake by the respective roots.

The differences in P uptake patterns between the rhizotron types were also reflected by the ^33^P activities within the roots, i.e., they truly resulted from P acquisition from the hydroxyapatite: Roots from sub-irrigated rhizotrons (Fig. 4a,c) displayed higher activities than from the top-irrigated ones (Fig. 4b,d). As we detected higher radioactive intensities in the roots at the bottom of the rhizotrons, surrounding the lower hydroxyapatite hotspots, we concluded that ^33^P was primarily acquired from the deeper hotspots at 30 cm depth, likely promoted by better subsoil moisture supply for P uptake provided via sub-irrigation. Potentially, root exudates and the resulting acidification of apatite surfaces were responsible for the release of P from apatite^28^, even if no overall change in soil pH was detected (data not shown), as the remaining apatite likely buffered all gross pH changes.

**Figure 5:**
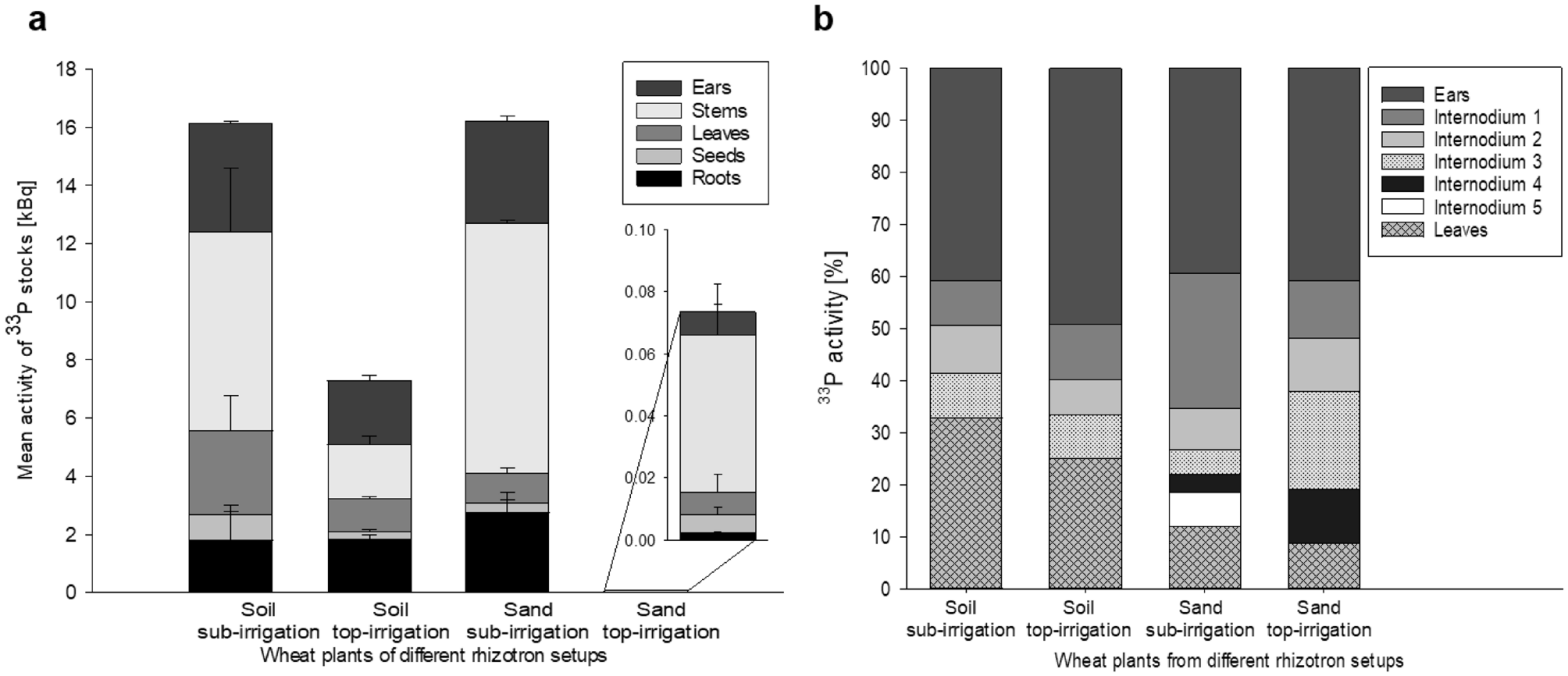
(**a**) Liquid scintillation counting measurements of different plant segments (n = 3) from the different rhizotron variants for the ^33^P stocks; (**b**) percentage distribution of ^33^P within wheat plants from soil and sand rhizotrons (n = 3) after 44 days.

For the quantitative assessment of ^33^P activities, we performed LSC measurements of digested plants and soils. The results confirmed the visual impressions. Clearly, higher ^33^P activities were found in wheat plants when grown under conditions of sub-irrigation than for plants grown under top-irrigation (Fig. 5a). The consistent pattern resulted from both, elevated P uptake and thus elevated ^33^P activity within the plants (Fig. S3; Supplementary Information), as well as from the improved overall plant growth in the variants with sub-irrigation (Table 1). Between the two soil rhizotron types, an increase of the ^33^P stock activity by a factor of 2.2 (54.9%) could be determined within the wheat plants, promoted by increased subsoil moisture contents, in comparison to the sand rhizotrons, where the increase was equal to a factor of 231.7 (99.6%) (Fig. 5a). These results underline the importance and beneficial effect of soil moisture for the accessibility and acquisition of subsoil P originally bound in hydroxyapatite.

When summing up the percentage distribution of the ^33^P radiotracer within the different plant segments, the activity was rather equally distributed within the wheat organs of the soil rhizotrons, whereas in the sand rhizotrons a gradient of increasing ^33^P accrual from the lowest internode to the ears was found (Fig. 5b). This data illustrates once more that P allocation in the plant is variable for this nutrient and water stress promotes the redistribution of nutrients from the roots to the ear.

## Conclusion

Wheat plants grown in soil rhizotrons grew similarly regardless of whether they were irrigated via the top- or subsoil; however, nutrient uptake differed. Overall, the plant acquired significantly more P from subsoil hydroxyapatite when the subsoil was moist. Subsoil water rather than topsoil water and related nutrient foraging thus control overall nutrient uptake from the subsoil. Additionally, our data shows that different irrigation may affect overall P uptake > fourfold and ^33^P uptake 231.7-fold within a given class of rhizotrons. Compared with the soil rhizotrons, elevated nutrient deficiency in the sandy ones reduced leaf formation, shortened the internodes and promoted a premature grain filling, which went along with an enhanced apatite-derived ^33^P uptake into the ears. Apatite-bound P is thus not recalcitrant but partially available to plants, even within one cropping season, but the degree of its utilization depends on the overall nutrient status and soil water distribution.

## Supplementary information

Supplementary information
