# Supplementary material for: Bioavailability and -accessibility of subsoil allocated 33P-labelled hydroxyapatite to wheat under different moisture supply
Source: Sci Rep. 2020 Oct 13;10:17140. doi: 10.1038/s41598-020-74225-3 (PMC7553936; doi:10.1038/s41598-020-74225-3)
Supplement: Supplementary file 1 — Supplementary information [file 41598_2020_74225_MOESM1_ESM.pdf]

Supplementary Information

of

"Bioavailability and -accessibility of subsoil allocated ^{33}P-labelled hydroxyapatite to wheat under different moisture supply"

Authors:

Jan Wolff[1,2,], Diana Hofmann[2], Maximilian Koch[2], Roland Bol[2], Andrea Schnepf[2], Wulf Amelung[1,2]*

[1]Institute for Crop Science and Resource Conservation (INRES) – Soil Science and Soil Ecology, University of Bonn, Nussallee 13, 53115 Bonn, Germany

[2]Institute for Bio- and Geosciences – IBG-3: Agrosphere, Forschungszentrum Jülich GmbH, 52425 Jülich, Germany

*Corresponding author:

Jan Wolff (E-mail: jwolff@uni-bonn.de, Tel.: +49 228 73 2981)

Institute for Crop Science and Resource Conservation (INRES) – Soil Science and Soil Ecology, University of Bonn, Nussallee 13, 53115 Bonn, Germany

Content:

Figure S1

Figure S2

Figure S3

Figure S4

Table S1

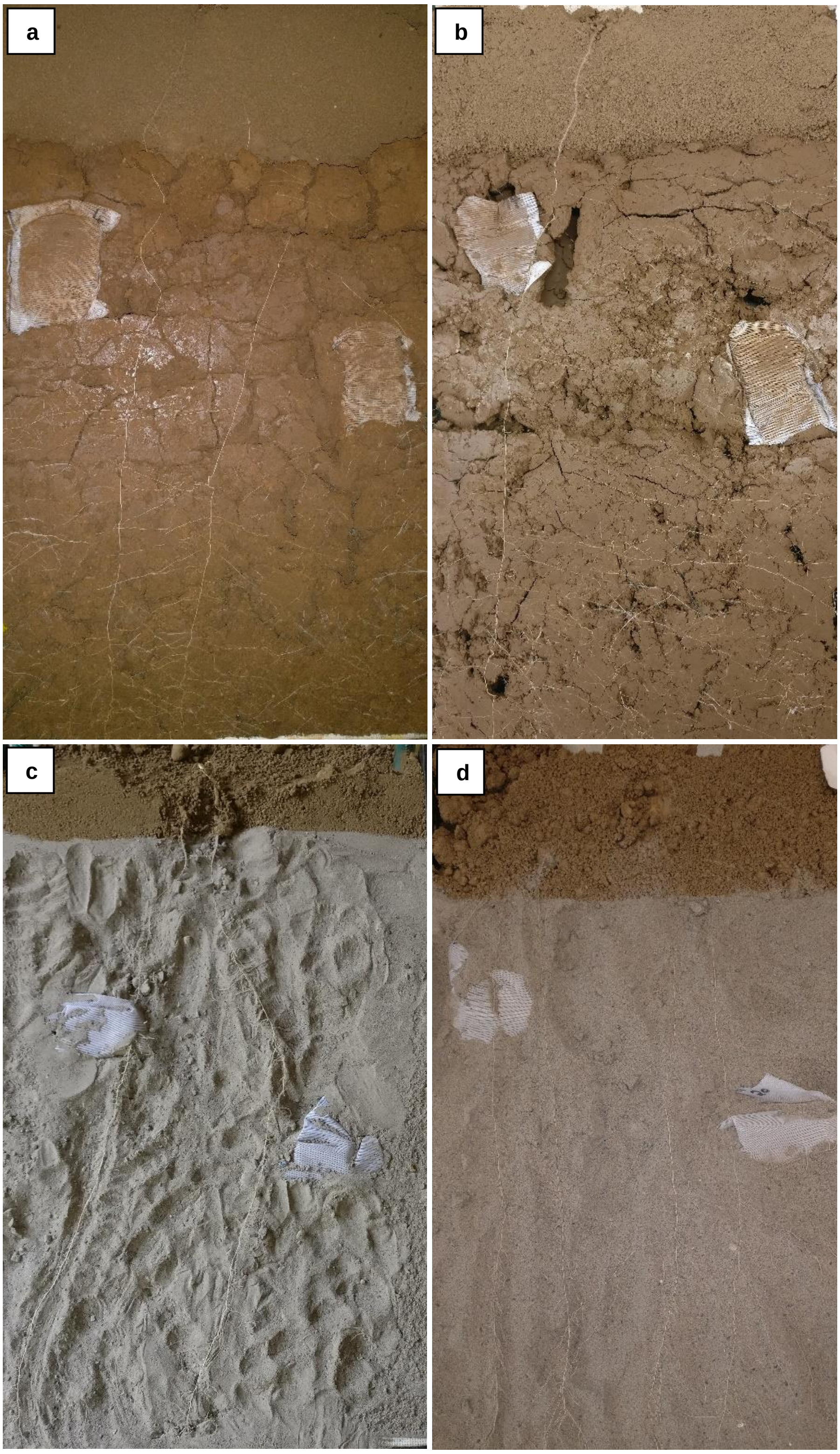

Figure S1: *Root growth within the four different rhizotron variants after 44 days, showing soil rhizotrons with* ***a)*** *sub-irrigation, and* ***b)*** *top-irrigation, as well as sand rhizotrons with* ***c)*** *sub-irrigation, and* ***d)*** *top-irrigation, respectively.*

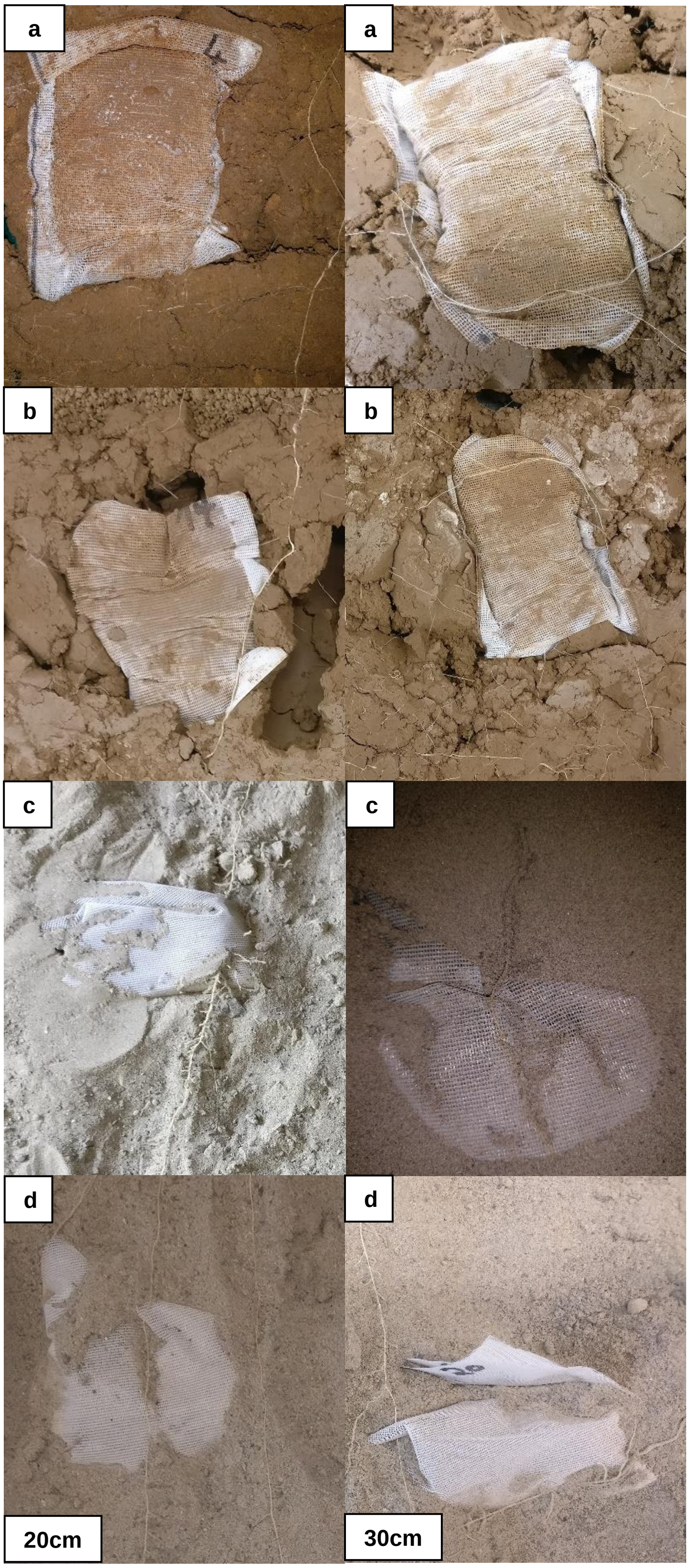

Figure S2: *Root growth close to and within* ^{33}*P hydroxyapatite hotspots within the different rhizotron variants after 44 days, showing soil rhizotrons with* ***a)*** *sub-irrigation, and* ***b)*** *top-irrigation, as well as sand rhizotrons with* ***c)*** *sub-irrigation, and* ***d)*** *top-irrigation. Left side = hotspots in 20cm depth; right side = hotspots in 30cm depth.*

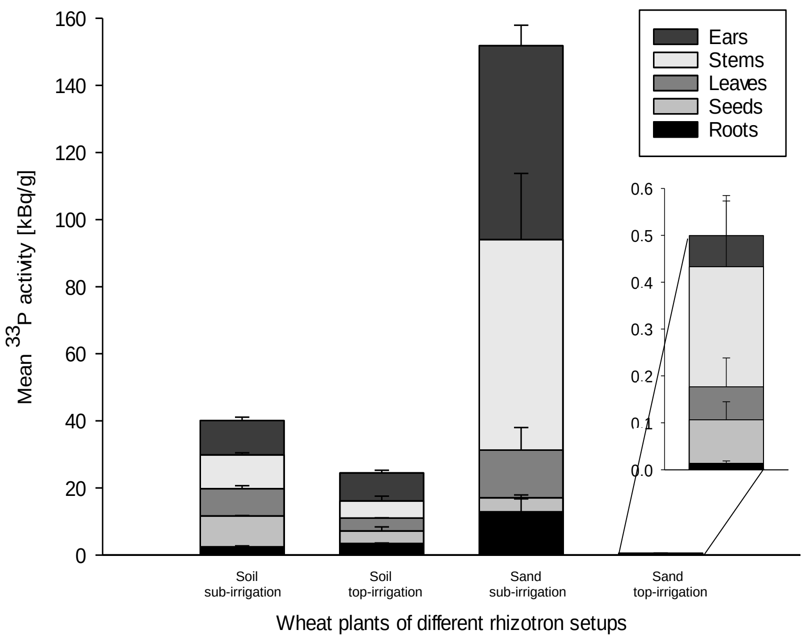

Figure S3: *Liquid scintillation counting measurements of different plant segments (n = 3) from the different rhizotron setups displayed as ^{33}P activity per gram. Sub-irrigation = irrigation via subsoil; top-irrigation = irrigation via topsoil.*

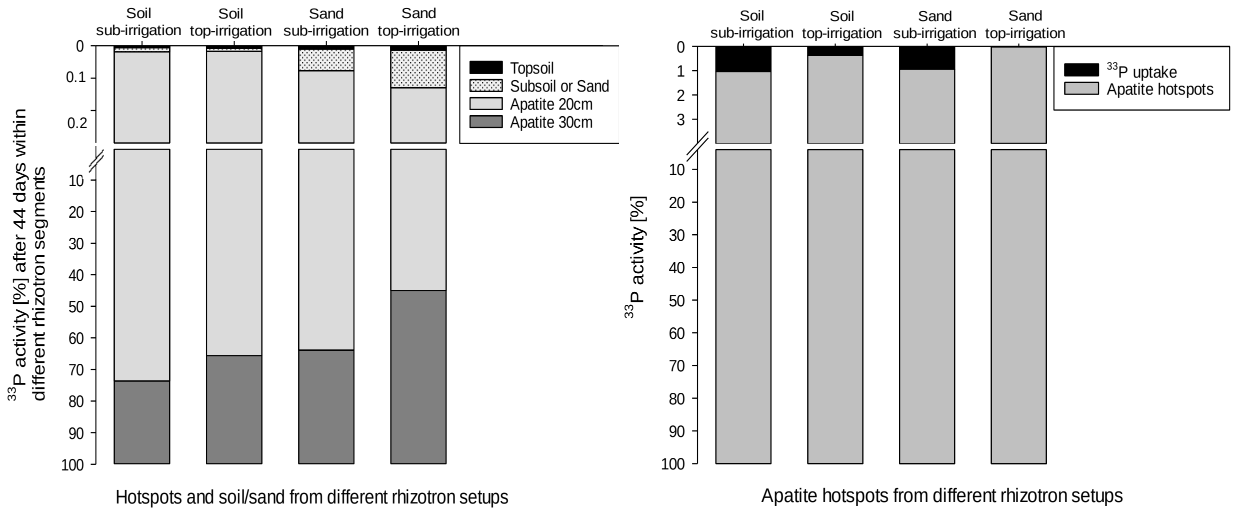

Figure S4: *Percentage distribution of ^{33}P in soils, sands and apatite hotspots within the different rhizotron setups remaining after 44 days (n = 3). The left Figure displays the ^{33}P activity in the topsoil and topsoil/sand in comparison with the implemented apatite hotspots; the right figure displays the remaining ^{33}P activity within the hotspots compared to the amount ^{33}P that has been taken up during the experiment. Sub-irrigation = irrigation via subsoil, i; top-irrigation = irrigation via topsoil.*

LSC measurements of the hotspots as well as the topsoil, subsoils and sands were carried out to detect possible leaching effects of the ^{33}P from the hotspots into the surrounding areas as well as to determine the amounts being taken up from the individual hotspots (Fig. S4 a and b). As can be seen, no ^{33}P could be detected outside the hotspots and, apart from the topsoil irrigated sand rhizotrons, the deeper, more moist located hotspots represented the preferred source for ^{33}P uptake. As already seen from the previous data, the equal distribution of activity in the hotspots of the topsoil irrigated sand rhizotrons indicates that almost no ^{33}P was taken up from these sources. Within the 44 days of the experiment, it was found that a maximum of 1% of the total amount of the ^{33}P was taken up with respect to hotspot activity, which could qualify hydroxyapatite as a possible natural long-term phosphorus source in agricultural subsoils.

Table S1: *Mg, K and Ca stocks of plant segments (n = 3) from different rhizotron trials.*

**Treatments**	**Soil Sub-irrigation**			**Soil top-irrigation**			**Sand sub-irrigation**			**Sand Top-irrigation**		
**Mg stocks**						**[mg]**						
Ears	0,18	±	0,01	0,19	±	0,01	1,02	±	0,14	0,19	±	0,04
Stems	0,37	±	0,01	0,47	±	0,08	2,11	±	0,07	0,98	±	0,05
Leaves	0,59	±	0,09	0,68	±	0,14	1,10	±	0,18	1,18	±	0,45
Seed	3,77	±	1,11	0,95	±	0,59	4,81	±	0,64	0,30	±	0,04
Roots	7,10	±	1,44	8,91	±	1,70	1,40	±	0,75	0,46	±	0,45
**Total**	12,00	±	0,75	11,20	±	1,05	10,43	±	1,03	3,10	±	0,68
**K stocks**												
Ears	3,25	±	0,24	3,39	±	0,16	9,78	±	1,87	1,07	±	0,96
Stems	14,78	±	2,31	14,40	±	2,60	22,43	±	7,72	8,00	±	1,34
Leaves	7,89	±	2,12	10,05	±	2,21	2,57	±	0,89	3,98	±	1,41
Seed	68,54	±	7,95	18,84	±	5,00	9,91	±	1,35	0,80	±	0,12
Roots	16,57	±	1,93	21,78	±	2,73	0,22	±	0,05	0,26	±	0,06
**Total**	111,04	±	7,48	68,45	±	6,94	44,90	±	10,64	14,11	±	2,95
**Ca stocks**												
Ears	0,23	±	0,01	0,27	±	0,03	2,08	±	0,29	0,32	±	0,19
Stems	0,72	±	0,08	0,89	±	0,08	7,79	±	1,04	3,81	±	0,36
Leaves	2,90	±	0,18	2,72	±	0,06	3,28	±	1,06	3,14	±	0,57
Seed	7,85	±	3,81	2,28	±	0,82	7,23	±	1,50	1,36	±	0,23
Roots	9,73	±	2,89	13,18	±	0,39	0,75	±	0,32	0,30	±	0,23
**Total**	21,44	±	1,88	19,34	±	0,50	21,13	±	1,38	8,93	±	0,80
